# Elevated Mercury Concentrations in Humans of Madre de Dios, Peru

**DOI:** 10.1371/journal.pone.0033305

**Published:** 2012-03-16

**Authors:** Katy Ashe

**Affiliations:** Department of Civil and Environmental Engineering, Stanford University, Stanford, California, United States of America; Centre National de la Recherche Scientifique, France

## Abstract

The enormous increase in practically unregulated mining in Madre de Dios Peru is leading to massive release of liquid elemental mercury to the environment. Rapidly increasing global prices for gold are causing a massive upsurge in artisanal mining in the Peruvian Amazon, considered to be one of the most biodiverse places on the planet. This study identifies the current levels of mercury in the human population, through identifying levels of total mercury in human hair in mining zones of Madre de Dios Department and in the nearby city of Puerto Maldonado. A regression analysis reveals that fish consumption, gender, and location of residence were significant indicators of mercury levels; while duration of residence and age had no significant relationship to mercury levels. Increased fish consumption levels were the strongest indicators of increased total mercury levels across the entire population. The levels of total mercury in hair was significantly (α = 0.05) higher in mining zones, than Puerto Maldonado. In both areas men had significantly higher levels than women, likely due to a difference in metabolism or varying levels of direct involvement in gold mining- a male predominated industry. This is the first study to show the health threat that mercury poses to this region, however further research needs to be done to gain a more refined understanding of the predominant routes of exposure in this population.

## Introduction

With global market prices of gold increasing at a rate of approximately 18% each year [1], poor migrants from different regions of Peru are flocking to the Amazonian department of Madre de Dios to find their fortune as artisanal gold miners. The rainforest in Madre de Dios, considered one of the most bio-diverse places on the planet [2], is rapidly being cleared to make way for mining camps. Forest conversion in the mining zones surrounding Puerto Maldonado has increased six-fold from 2003–2006, leading to a current mining-related deforestation rate of 1,915 hectares annually. Unfortunately, deforestation rates show no signs of slowing [3].

The Madre de Dios Department of Peru produces approximately 70% percent of Peru’s artisanal gold production (Ministry of Energy and Mines 1998 *in* [4]). As many as 30,000 miners are estimated to be working in this area [5], although exact numbers are difficult to determine because Madre de Dios also has the highest number of unapproved mining permits in the country [6]. The number of illegal miners in the region is unknown, but aerial photographs of rapidly expanding mining zones indicate that the number is dramatically increasing [3]. Technically, the approval of a mining permit necessitates an environmental impact report. Practically, the low level of enforcement leaves little incentive to apply. The lack of regulation leaves limited opportunity for government instruction on safe mercury handling practices. This inadequate knowledge of mercury use is leading to its haphazard handling and inevitable release to the environment.

Artisanal mining worldwide is responsible for one third of all mercury released into the environment – approximately 1000 tons/year [7]. As of 2009 Peru was importing mercury at a rate of 130 tons per year [8], over 95% of which is being used directly in artisanal gold mining within the country (Superintendencia Nacional de Aduanas del Peru *in* [4]). This creates an enormous potential for mercury exposure to miners themselves and those living within the surrounding area. The location of Madre de Dios at the headwaters of the Amazon basin creates a potential transportation and diffusion of this environmental health problem throughout a major portion of the Amazon River Basin and ultimately the world’s oceans.

Excess mercury being dumped into the environment and transported via the atmosphere, waterways, and sediment provides the possibility for widespread exposure to mercury. During gold processing miners generally come into direct dermal contact with mercury by mixing the amalgam while barefoot and handling the liquid mercury with bare hands. As the mercury is heated from the amalgam over an open flame, the vapor is inhaled by shopkeepers and those in the vicinity. The incidence of inhalation is further increased by the superstition that, in order to prevent the loss of gold, one must stand in close proximity to the amalgam. Many other misunderstandings of mercury contamination, not based in scientific fact, also contribute to exposure. As an example, it is widely believed in mining zones that mercury can only enter the body when in direct contact with open wounds, and that liquid mercury can be safely used as a laxative. Miners are directly exposed to mercury through dermal exposure, inhalation of vapor, and intake of animal products that contain high levels of methyl-mercury. The general population is predominantly exposed through the latter two pathways.

Little is known in Madre de Dios about the degree to which mining practices are currently affecting human health in the region. Mercury is known to have detrimental effects on human health, but at present there is little empirical data to suggest that actual current impacts on the region’s residents. This study is the first intended to provide some rigorous scientific data on the levels of mercury in the population of Madre de Dios. Additionally, we will assess how several of the main risk factors including location of residence, duration of residence, sex, fish intake, and age impact the levels of mercury.

## Materials and Methods

### 1. Study Area

The Madre de Dios Department is located in the lowland rainforest of Southeastern Peru, bordering Brazil and Bolivia. Because Madre de Dios is predominantly covered in rainforest, the highest population density in this department lies in the city of Puerto Maldonado, located at the confluence of the Madre de Dios and Tambopata rivers. Puerto Maldonado is connected to Cusco via the Interoceanic Highway, which serves as the main route for transportation in this region.

Though annual gold exports from this region are difficult to quantify, in 2009 they were estimated at16 tons [5]. However, with the most recent mercury imports at 175 tons per year, this value should be dramatically higher at present [1]. Gold mining is one of the primary economic activities in this region, though it is hard to track due to the lack of centralized organization [1]. Mining operations in this region primarily consist of small riverine or terrestrial operations.

Along the Interoceanic Highway terrestrial mining zones are rapidly manifesting and expanding in the rainforest. Small bodies of water, such as a small swamp, are found to contain a sufficient level of gold to support prospecting mining – other settlers soon follow. These small mining camps initially consist of a few families who clear the surrounding primary rainforest in order to make space for more miners and their rigs; these mining zones eventually transition into sprawling shanty towns. Due to the illegal nature of this type of mining, these zones are typically located several miles from the road in order to prevent access by government officials. These zones have little central organization and are seeing an exponential influx of settlers, primarily young people from the Andean Highlands [1].

Samples for this study were collected in six mining zones, which at the time of the study were going by the self-reported names of Nueva Arequipa, Alto Libertad, Kilometro 108 (named for distance from Puerto Maldonado on the Interoceanic Highway), Kilometro 102, Huepetue, and Lamar (or Kilometro 102.5). These mining zones were analyzed as a single generic “mining zone” population for the purpose of this study. This is a reasonable assumption due to the extreme geographic proximity of these zones and the shared culture between the communities. The Puerto Maldonado population was sampled within the city limits and predominantly in La Joya neighborhood.

### 2. Sampling and Survey

Human hair samples were collected from all study participants, who were also asked to complete a verbally delivered health survey. Hair samples of approximately 0.5 grams were collected from close to the occipital area of the scalp with stainless steel scissors and stored in double plastic bags at room temperature.

Participants were asked to provide their age, sex, zone of residence, duration of residence in the zone, and monthly number of times they consume fish. The brevity of the survey and small hair sample size minimized the study’s social impact in collection zones, a key component in retaining safety of all parties involved. Due to the illegal nature of mining and recent controversy surrounding the occupation, the subjects were approached with the utmost care and awareness of potential risks. Because of the concern for safety, no inquiry about the specific occupation of each individual was made. This inquiry would have put the study, those participating in the study, and the researcher conducting the interviews at potential risk. Therefore, the two populations addressed in this study were named according to their geographic regions – as people living in mining zones (determined to be predominantly in the occupation of mining) and those living in the city center of Puerto Maldonado (some occupational miners, however predominantly not directly involved in mining).

Puerto Maldonado is not a control group for mercury exposure due to the high levels of mercury vapor from some urban gold shops, and occasional occupational miners that live within the city limits. However, individuals living in mining zones are predominantly involved in gold-mining; while people from Puerto Maldonado are less likely to be occupied in the trade. Unfortunately, information on occupation was not attainable without risk of personal and participant safety due to the illegal nature of gold-mining. Therefore, a comparison between mercury concentrations in Puerto Maldonado and Mining Zone populations is performed in order to better understand the mercury exposure experienced given the difference in physical environment and typical occupation. The total number of participants sampled was 204, with the breakdown of location and gender shown in Table 1.

**Table 1:** Demographics of participants in the study.

Location of Residence	Gender	n
Puerto Maldonado	Men	60
	Women	44
Mining Zones	Men	38
	Women	62

### 3. Chemical Analysis

Hair samples were transported and analyzed in a mercury-safe laboratory on Stanford University campus. The hair samples were washed with 20.0 mL of an EDTA 0.01% solution and dried at 40°C. Details of extraction of hair samples followed the procedure outline as the guideline for standard procedure of use for the *Tekran Series 2600 Total Mercury System* [9].

The total mercury concentrations in hair were analyzed by Cold Vapor Atomic Fluorescence Spectrometry using the Tekran Series 2600 as detail in EPA Method 1631. Analysis of reagent blanks and reference standards were used to assure analytical control.

### 4. Statistical Analysis

All reported values from the sample populations were represented by the box plots. Hair mercury levels underwent a logarithmic transformation to improve normality before further analysis. The values represented with the boxes are the first quartile (lower hinge), median, and third quartile (upper hinge). The whiskers are the upper and lower adjacent values as defined by Tukey [10]. The points represented by nodes outside of the box plots are individual values that are outside of the range of adjacent values. Significance of sample differences was tested using a two-sample mean-comparison t-test with unequal variances to a 95% confidence level.

A generalized linear model (GLM) for Gaussian data using maximum quasilikelihood optimization of a linear model was fit to the logarithmic-transformation of all mercury hair levels. This model was used to determine the relative impact of age, residence location, duration of residence, gender and fish consumption on determining total hair mercury levels [11]. Statistical analyses were performed using Stata 11 software [12].

## Results

Using the GLM framework described above we found that fish consumption levels, gender, and residence location (arranged in decreasing significance) were all significant (α = 0.05) predictors of the mercury levels found in the hair samples (see Table 2). The age and duration of residence in a particular region had no significant relationship to the mercury levels measured in the hair.

**Table 2:** Observed relationships of dependent variables to logarithmic-transformed total mercury levels found in the hair samples using a generalized linear model.

Dependent Variable	z	P**>** | z |
Age	–0.18	0.8543
Gender	–3.05	0.0023
Residence Location	2.86	0.0042
Duration of Residence	–0.18	0.8582
Monthly Fish Consumption	3.7	0.0002

### 1. Residence Location

**Figure 1:**
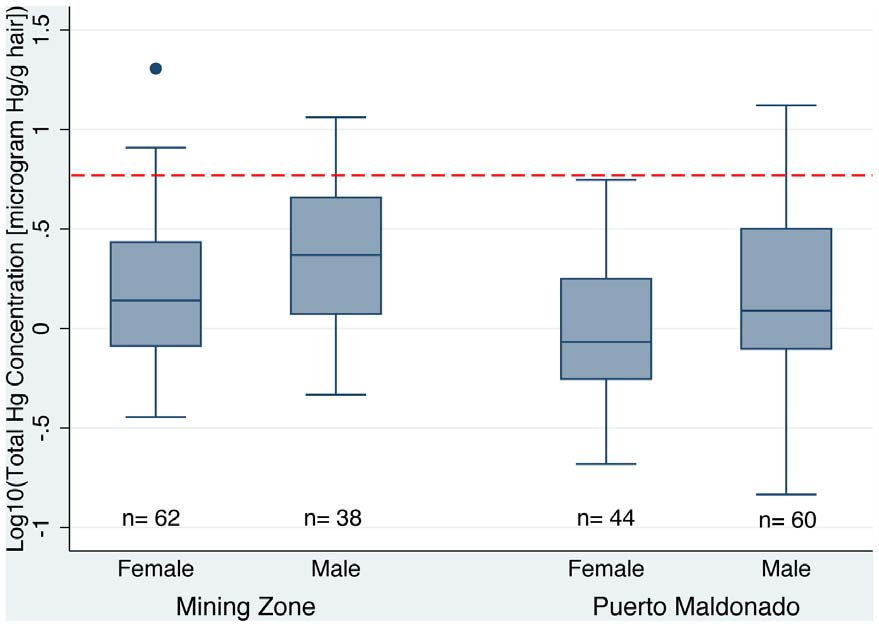
Mercury levels in hair samples by location of residence and gender. Red line indicates the level of mercury in human hair considered to start unhealthy symptoms by WHO (6 µg Hg/grams of dry hair) [13].

Location of residence is significantly (α = 0.05) related to the total mercury levels we measured in hair. As expected, mercury levels in hair of people living within mining zones were found to be significantly (α = 0.05) higher than people living in Puerto Maldonado (see Table 1 & Figure 1). The men living in mining zones had significantly (α = 0.05) higher levels of mercury than men in Puerto Maldonado. Likewise, the women in mining zones had significantly (α = 0.05) higher levels of mercury than women in Puerto Maldonado. Mercury levels above 6.0 µg Hg/gram of dry hair start unhealthy levels, while levels above 16.0 µg/g are considered toxicologically symptomatic by World Health Organization (WHO) standards [13]. In mining zones, the observed percentage of sample population having unhealthy mercury levels was 11%, while it was only 5% in the city-dwelling population. Toxicologically symptomatic levels were only witnessed in the mining zones as shown in Table 3 [1].

**Table 3:** Number of unhealthy and toxicologically symptomatic levels of Hg detected in human hair [12].

Location of Residence	Gender	n	Number with unhealthy levels of Hg	Number with toxicologically symptomatic levels of Hg
Puerto Maldonado	Men	60	5 (8%)	0 (0%)
	Women	44	0 (0%)	0 (0%)
Mining Zones	Men	38	7 (18%)	0 (0%)
	Women	62	4 (6%)	1 (2%)

The main reason for the significant difference mercury levels in these populations likely due to the larger proportion of miners that live in the mining camps, who have a greater probability of exposure to mercury. Higher levels present in the hair of people living in mining zones may be attributable to the higher density of Hg-vapor producing gold-shops within the camps, which have been shown to provide significant risks to Amazonian artisanal mining populations [14]; higher levels of mercury in water; and/or dust within mining zones may also be leading to the higher levels of exposure.

### 2. Sex

Gender was the second most important risk factor with respect to mercury exposure analyzed in this study. Males had significantly (α = 0.05) higher levels of mercury than women in the population (see Figure 1 & Table 1).

Men in the mining zones had the highest levels of mercury of any demographic. Within these mining zones the men had an average of 3.39 µg/g, while women have an average of 2.23 µg/g. The percentage of the unhealthy levels of mercury was 18% and 6%, respectively.

The same gender trends were seen in the Puerto Maldonado population; men had an average of 2.30 µg/g and women had an average of 1.37 µg/g. There were no unhealthy levels of mercury witnessed in the population of women sampled in Puerto Maldonado. The incidence of unhealthy levels of mercury in men was 8%, which is even higher that the incidence rate of women living in the mining zones.

### 3. Fish Intake

**Figure 2:**
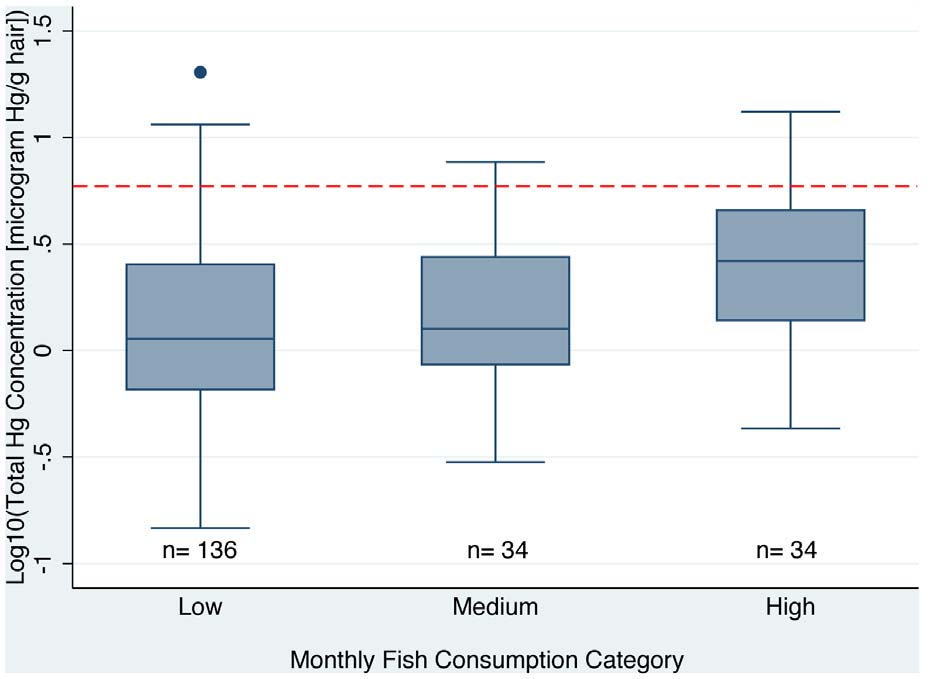
Total mercury levels in hair samples by fish consumption levels. Red line indicates the level of mercury considered unhealthy by WHO [13].

Monthly fish consumption levels were the most significant predictor of mercury hair levels of the parameters explored in this study using the glm framework (see Table 1 and Figure 2). Intake was measured as the number of times a person reported eating a fish meal each month.

Fish consumption was broken into three qualitative categories of fish consumption for simpler analysis: 0–5 (low), 6–11 (medium), and 12 or more (high) fish meals consumed each month. The average levels of mercury in each group were 2.02, 2.12, and 3.49 µg/g, respectively. People that were in the lower two fish consumption levels (0–5 and 6–11 fish meals per month) did not have significantly different levels of mercury in their hair (α = 0.05). People that ate 12 or more fish meals per month had significantly higher levels compared to the other two groups. The unhealthy levels of mercury were 7% and 6% for the lowest two consumptive groups. Of the people that reported eating more than 12 fish per month, 18% had unhealthy levels of mercury in their body. Cultural eating habits can often influence fish consumption levels, however for those surveyed there were no significant (α = 0.05) difference of fish consumption found across age, gender, or location.

## Discussion

The strongest predictor of total mercury levels found in hair samples was the amount of fish consumed by that individual. This is unsurprising, as levels of fish consumption have been shown in numerous previous studies, including those in Amazonia, to have a positive correlation with the total and methyl mercury levels found in a population. Additionally, across the populations that have been studied in Amazonia, a clear pattern emerges illustrating that communities with much higher fish consumption have much higher mercury levels (see Table 4). Mercury bio-accumulates and generally biomagnifies as one moves up the food chain.

**Table 4:** Previous studies on Total Hg in human hair [µg Hg/g dry hair] in different countries for comparison [15]–[22].

Country	State	Region	n	Mean	Range	Fish Intake	Population Characteristics	Study
USA	Minnesota, Wisconsin, Illinois, Indiana, Ohio, Michichigan	EPA Region V Great Lakes area	182	0.30	(0.01–3.51)	Low	General population	Pellizzari 1999 [15]
Japan	Minamata, Kumamoto, Tottori, Wakayama, Chiba	All	3686	1.96	(0.00–26.76)	High	General population	Yasutake 2003 [16]
Brazil	Pará	Santana do Ituqui	321	4.33	(0.40–11.60)	High	Small riverside community	Santos 2002 [17]
Brazil	Pará	Aldeia do Lago Grande	316	3.98	(0.40–11.76)	High	Small riverside community	Santos 2002 [17]
Brazil	Pará	Tabatinga	499	5.37	(0.37–16.96)	High	Small riverside community	Santos 2002 [17]
Brazil	Pará	Caxiuanã	214	8.58	(0.61–45.59)	High	Small riverside community	Santos 2002 [17]
Brazil	Rondonia	Madeira River	241	17.2	303	High	Riverside fishing community	Barbosa 1995 [18]
Brazil	Rondonia	Madeira River	75	8.7	31.9	High	Riverside community from Ecological Cuniã Reserve	Barbosa 1995 [18]
Brazil	Amazonas	Negro River	76	21.4	(1.66–59.01)	High	Small riverside community	Barbosa 2001 [19]
Brazil	Bahia	Barreiras	76	16.4	(1.8–53.8)	High	Fishermen and their families	Harada 2001 [20]
Brazil	Pará	Sao Luiz do Tapajos	44	20.8	(5.1–42.2)	High	Fishermen and their families	Harada 2001 [20]
Brazil	Pará	Tucuruí area	125	35	(0.9–240)	High	Communities living around a hydroelectric dam reservoir	Leino 1995 [21]
Brazil	Pará	Fresco river	419	8.0	37	Medium	Kayapó Indians	Barbosa 1995 [18]
Brazil	Amazonas	Balbina Reservoir	53	6.54	(1.2–22.0)	Medium	Fish is one of the main source of protein	Kehrig 1998 [22]
Brazil	Pará	Maria Bonita	145	3		Low	Gold prospectors (Garimpeiros)	Barbosa 1995 [18]
Peru	Madre de Dios	Puerto Maldonado	104	1.90	(0.15–13.22)	Low	City dwellers, some gold prospectors	This Study
Peru	Madre de Dios	Mining zones near Puerto Maldonado	100	2.67	(0.36–20.26)	Low	Gold prospectors and others in mining camps	This Study

However, increased fish consumption does not always lead to increased mercury concentrations as mercury concentrations across fish species in a region are not uniform. For example, a study conducted on fishermen in the Brazilian Amazon found that only the subpopulation eating primarily carnivorous fish species had mercury levels that significantly correlated to levels of fish consumption [21]. The data collected for fish consumption in this study did not detail the trophic level or species typically eaten. In the future, mercury exposure surveys should hone in on the fish consumption patterns in this region at greater detail to better access this primary risk factor.

Gender emerged as the second strongest predictor of mercury levels in this population. The difference in mercury levels across the gender divide can partially be attributed to differential metabolism and exposure to this toxin. Physiological factors, like gender, have been shown to influence how toxins are metabolized [19]. Some studies on populations in the Amazon have found that women typically have lower levels of mercury [19] [23], especially women of reproductive age (16–40 years) who have substantially lower levels of mercury due to pregnancy and lactation [24]–[25]. Our findings corroborate the significantly lower levels found in the women of Amazonian populations. These lower levels could be explained by the protective metabolism of females or the cultural drivers of exposure in this population. Gold mining is typically a male occupation in this region; however some women do work in the gold shops that heat the mercury off of the amalgam. Unfortunately, this survey did not collect data on the specific occupations of the participants; therefore the relative role that occupational exposure and protective metabolism play cannot be definitely shown. There was no significant relationship between gender and fish consumption, so dietary habits are most likely not leading to the difference in mercury levels between genders.

Location of residence (i.e. either Puerto Maldonado or one of the surrounding mining zones) was a significant risk factor for the mercury levels found in this population. This is unsurprising, as the occupational and environmental exposure within the two environments is substantially different. Within the mining zones the vast majority of the population is directly exposed to mercury through the process of mining or refining gold. Additionally, the mining zones house a much higher density of gold shops that are wafting mercury vapor into the air and artisanal mines that are leaking mercury into water bodies located in the direct vicinity of the community. These aforementioned exposures of mercury are not unheard of in Puerto Maldonado; however they are sparse in comparison. Based on the varying magnitudes of exposure, it is unsurprising that the residents of Puerto Maldonado had significantly less total mercury in their hair. Unfortunately, data was not collected on levels of methyl mercury in the hair samples, so the relative presence of mercury species is unknown. Occupational exposure to mercury was shown in previous study of artisanal gold miners in Brazil to lead to a wildly varying and occasionally much higher percentage of the less toxic, inorganic mercury [26]. So, despite the significantly higher level of total mercury within residents of the mining zones; the increased health risks are hard to definitively determine.

Contrary to our study, a previous study conducted in the Amazon found the population within a mining zone to have substantially lower levels than a neighboring native tribe living in the same region [18]. However, the gold mining population in the aforementioned study consumed very little fish, while it was one of the main sources of protein for the local tribe [18]. Fish consumption patterns did not vary significantly between the two locations of residence for our study and may mark the main difference between these two studies.

Age was not significantly correlated with mercury levels as has been shown in some previous Amazonian studies [19] [27]. Variation of mercury across age is often attributed to shifting cultural fish consumption patterns between generations [16]. This resonates with our finding of no correlation between age and mercury level; in unison with no significant correlation found between fish consumption and age.

Overall, the mercury levels found in this study are characteristic of previous studies in the Amazon that focused on populations where fish consumption was not a major source of protein. However, this study found much lower levels than most previously studied populations in the Brazilian Amazon, probably due to the fact that majority have focused on small riverine fishing communities that utilize fish as the major source of protein in their diet (see Table 4). It is important to remember that our study does not fully characterize the entire population of Madre de Dios, rather a subset of the population dwelling in the capitol city and mining zones along the Interoceanic highway. As mercury exposure in this region is further investigated it will be vital to study the riverine communities that predominantly subsist on fish.

### Conclusion

Mercury contamination from mining has taken place in the Peruvian Amazon since the time of the Incas [4]. The release of this persistent toxin is occurring at historically unprecedented rates with projected future increases as mercury imports escalate [3]. Mercury contamination in the Madre de Dios is creating health impacts that are affecting populations in the mining zones and in the neighboring regions alike. However, the full effects of mercury-related health issues have yet to be seen; as the this persistent toxin continues to accumulate in the natural environment we should expect to see levels of mercury.

This study underlines the fact that unhealthy levels of mercury are prevalent in this region. Fish consumption levels, location of residence, and gender are significantly related to total mercury levels found in this populations hair; however further work could be done if we want to better understand these relationships and how they evolve as more mercury continues to enter the environment. Hopefully, as we move toward finding a solution to this contamination issue a scientifically informed conversation can take place that will take into account the full extent of negative externalities of the practice of artisanal mining; including the threats mercury poses to current and future generations of the Department of Madre de Dios.
